# Network Design for Municipal Solid Waste Collection: A Case Study of the Nanjing Jiangbei New Area

**DOI:** 10.3390/ijerph15122812

**Published:** 2018-12-10

**Authors:** Jing Liang, Ming Liu

**Affiliations:** 1 School of Biology and Environment, Nanjing Polytechnic Institute, Nanjing 210048, China; 2 School of Economics and Management, Nanjing University of Science and Technology, Nanjing 210094, China; liuming@njust.edu.cn

**Keywords:** municipal solid waste, location and allocation, network design, mixed integer nonlinear programming, optimization

## Abstract

Garbage collection is an important part of municipal engineering. An effective service network design can help to reduce the municipal operation cost and improve its service level. In this paper, we propose an optimization model for the network design of municipal solid waste (MSW) collection in the Nanjing Jiangbei new area. The problem was formulated as a mixed integer nonlinear programming (MINLP) model with an emphasis on minimizing the annual operation cost. The model simultaneously decides on the optimal number of refuse transfer stations (RTSs), determines the relative size and location for each RTS, allocates each community to a specific RTS, and finally identifies the annual operation cost and service level for the optimal scenario as well as other scenarios. A custom solution procedure which hybrids an enumeration rule and a genetic algorithm was designed to solve the proposed model. A sensitivity analysis was also conducted to illustrate the impact of changes in parameters on the optimality of the proposed model. Test results revealed that our model could provide tangible policy recommendations for managing the MSW collection.

## 1. Introduction

This research was motivated by the practical concern of municipal solid waste (MSW) collection operations in the Nanjing Jiangbei new area. The new area, established on 27 June 2015, is located in the north of the Yangtze River in Nanjing and includes the districts of Pukou, Dachang, Liuhe, and Baguazhou Street of Qixia District. It is the cross point of the Yangtze River economic zone and the eastern coastal economic zone. Along with the rapid urbanization and the increasing population in the past three years, more and more garbage needs to be addressed. Nowadays, how to collect and dispose of the increasing amounts of MSW has become an urgent problem. This concern provides a good chance to use the operational research (OR) method to help local managers address the problem.

To investigate the existing approaches regarding MSW collection, we searched the related literature from multiple channels including Web of Science, Elseviser, and Google Scholar. We used keywords such as ‘solid waste collection’, ‘location and allocation’, ‘solid waste transfer-station location’ and found more than 100 related studies on this topic. We noticed that although research into MSW collection has been conducted for several years, most of them were published in environmental or scientific journals. Despite its importance and particularity, only a few studies have used the OR method to improve the municipal service level. In what follows, we briefly introduce some of the methods that we believe are most relevant to our study.

First, with the increasing population, the rapid growth of the economy, and the rise in living standards [1], more and more scholars have noticed the importance of MSW management in city living [2]. For example, reference [3] provided several solutions for solid waste management in a rapidly urbanizing area in Thailand. Reference [4] proposed a method for forecasting MSW generation in a fast-growing urban region with system dynamics modeling. Reference [5] analyzed some economic factors and political factors in influencing the privatization of MSW collection. Reference [6] investigated more than thirty cities in 22 developing countries across four continents to find the factors influencing the performance of waste management. Although these scholars pointed out that the capacity design of refuse transfer station (RTS) is an urgent problem because the current sites are seriously overloaded, they focused on the influencing factors regarding waste management, but did not design an optimal network for MSW collection.

Second, early scholars developed several optimization models to guide the location decisions of RTS. For example, Reference [7] proposed a general mathematical programming approach with four stages to determine the locations of RTSs. Reference [8] provided an analytical model and a traditional location-allocation model to determine the number, capacities, and locations of RTSs in an urban region. Reference [9] described the MSW problem as a network flow problem and developed a special purpose algorithm to solve it. Reference [10] presented a multi-objective optimization model for locating RTSs. The model sought the best tradeoff between minimizing costs and public opposition. Considering the inherent uncertainties in both economic and environmental goals, Reference [11] proposed a fuzzy goal programming approach to determine the best planning of MSW collection. Although early scholars have proven that resources used for MSW collection can be utilized more effectively if waste is transported to the disposal area via a RTS, the optimization model they used were deterministic and discrete, but not continuous.

Third, in the past five years, the optimization problem of MSW collection has attracted more and more attention due to quick urbanization and population growth. Reference [12] used an integer programing approach to optimize the route of MSW collection. The route solutions generated by the proposed methodology performed significantly better than the traditional routes that were manually designed. In reference [13], the vehicle routing problem (VRP) for MSW collection was complicated by inter-arrival time constraints. Reference [14] proposed a spatial allocation model to improve the MSW collection in Singapore. Reference [15] formulated MSW collection as a vehicle routing model within time windows, and applied their model in Danang City, Vietnam. Reference [16] proposed a bi-objective mixed integer optimization model to determine the locations of landfills and RTSs simultaneously, where one objective was the usual cost minimization, while the other minimized pollution. Unlike traditional approaches that always focus on RTS location, reference [17] surveyed the landfill location models that have appeared in the literature during the last forty years. In particular, reference [18] reported on a related literature review regarding operations research in MSW collection. Note that the majority of existing approaches have preferred to explore the VRP in MSW collection in recent years. Such an operation is tactical decision-making while network design for MSW collection is strategic decision-making. We also note that several existing approaches characterize MSW collection problem as a multi-source Weber problem (MWP) [16] or set cover problem (SCP) [13] on a continuous space. As is widely known, the number of new facilities to be opened is known in advance in a MWP and it is totally undetermined in a SCP. Unlike these two extremes, we preset a maximum number of RTSs that could be constructed in this paper. We tested all the possible scenarios and then determined the optimal number of RTS. Therefore, our model can be addressed as a middle state between the above two extremes.

Regarding the solution algorithm, reference [19] provided a heuristic approach combined with life cycle assessment to optimize MSW collection in Peru. Reference [20] presented a dynamic location analysis method to determine the optimal MSW collection strategy. Reference [21] designed the location-allocation problem for waste management by using the genetic algorithm (GA). Reference [22] used a Tabu search algorithm to design the optimal network for MSW collection. In particular, Reference [23] proposed three heuristics (GA, simulated annealing, and relocation search) to solve a location-allocation problem on a continuous space. The results demonstrated that no single algorithm dominated another. In other words, the three heuristics were well-matched. The above studies demonstrate that several algorithms are available to solve the optimization model for MSW collection.

**Figure 1:**
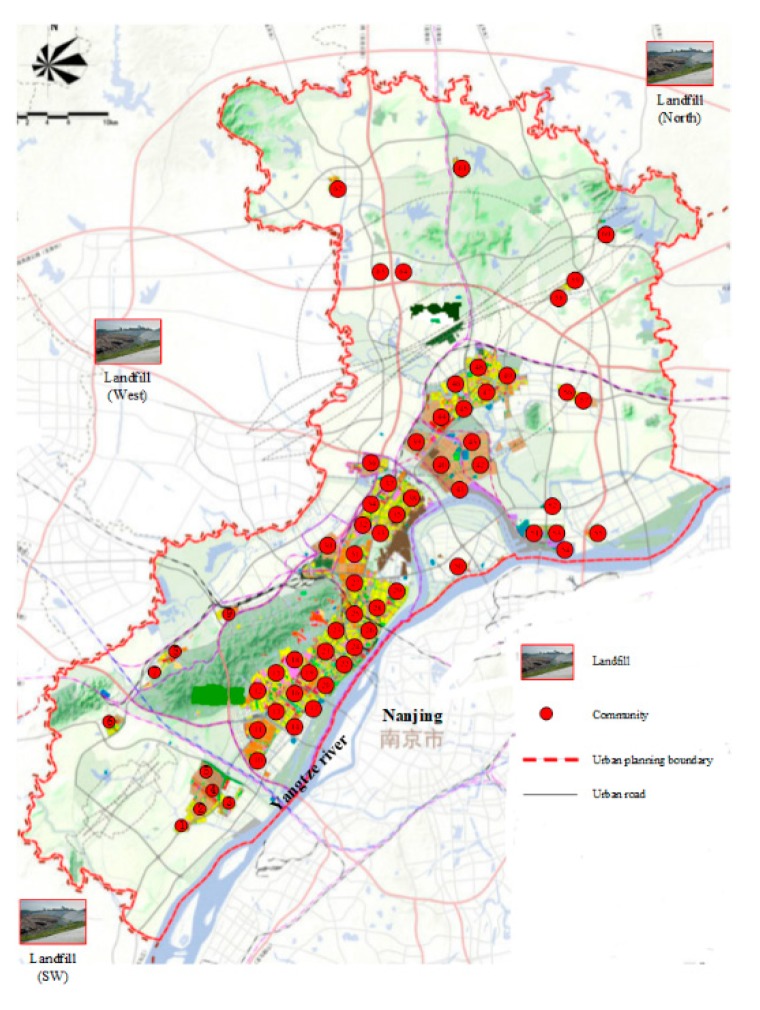
Population distribution and landfills in the Nanjing Jiangbei new area.

In this paper, we conducted a MSW collection in the Nanjing Jiangbei new area. Figure 1 shows that people in this new area are mainly concentrated in 64 communities including residential areas, commercial institutions, schools, colleges, industrial firms, and so on. There are three landfills, which are respectively located at the southwest, west, and north boundaries of Nanjing. The core questions in designing the MSW collection network includes: (1) how many refuse transfer stations (RTSs) should be constructed; (2) where to locate them; (3) what is the relative capacity of each RTS; (4) how to allocate the 64 communities to these RTSs; and (5) what is the annual operation cost and service level for the optimal scenario as well as other scenarios.

The above problems (1), (2), and (4) are commonly addressed in location-allocation problems. Generally, location and allocation for multiple facilities on a continuous space rests on the multi-source Weber problem (MWP), where the number of new facilities to be opened is known in advance. However, determining the number of RTSs is one of the main questions that need to be answered in the Nanjing Jiangbei new area. Therefore, our model needed to simultaneously decide how many RTSs should be opened and where to locate them. More important, we defined a relative size for each RTS and considered the optimal capacity setting for each of them (Question (3)). We tested the annual operation cost and service level for all possible scenarios, and finally determined the optimal scenario (Question (5)). The above two works make our paper different from the existing approaches, which have always preset several constraints for the RTS.

The rest of this paper is organized as follows. We provide details of the model formulation in Section 2. Section 3 designs an algorithm to effectively solve the optimization model. In Section 4, we apply the model to the Nanjing Jiangbei new area and provide a sensitivity analysis to examine the impact of changes in the model parameters on the optimality of the proposed model. Finally, our conclusions and suggestions for future research are presented in Section 5.

## 2. Model Formulation

### 2.1. Definition for the Relative Parameters

#### 2.1.1. Distance

In most location-allocation problems, distance is an indispensable variable because the core of optimization pursues that the sum of distance from the facility to the customers is minimized. Among the many measures proposed to determine the proximity between two points on a plane, the Euclidean distance is the simplest and easiest to implement [23]. For a RTS at (x,y) and a community l at $\left(a_{l},b_{l}\right)$, the Euclidean distance is computed as follows:

(1) $$d_{l}(x,y)=\sqrt{{\left(x-a_{l}\right)}^{2}+{\left(y-b_{l}\right)}^{2}}$$

The Euclidean distance between the landfill and the RTS can be similarly calculated. Note that we did not know how many RTSs should be constructed, and we did not have any potential locations for them in advance. In other words, the continuous map of the Nanjing Jiangbei new area provides infinite possibilities for the coordinates of each RTS.

#### 2.1.2. Unit Transportation Cost

In practice, there are two commonly used shipping methods: the full-truckload (TL) and less-than-truckload (LTL). By TL, we refer to the shipment of a full truckload of solid waste, and by LTL, we refer to shipments with a relatively small freight that do not fill the truck. According to our survey, the local government uses the TL mode to ship the processed solid waste from the RTSs to the landfills to ensure efficiency, and use the LTL mode to ship from the communities to the RTSs to satisfy the variable demand in different communities. Generally, the unit transportation cost is calculated on the basis of kilometers and the weight of the shipment. The price is expressed in terms of load distances, such as ton-kilometer, where a ton-kilometer is the amount of transportation activity to move a ton of solid waste over a distance of one kilometer. With regard to the speed, truck drivers are expected to transport freight at an average rate of 30 km per hour all the way to the destination.

#### 2.1.3. Demand Forecasting in the Communities

Assume that $n_{i}$ is the number of people who live in community i. q is the daily output of solid waste for each person. Generally, it falls in the range of (0.8, 1.2) kilograms. $\lambda_{i}$ is a coefficient to reflect the variation in consumption level. Let $D_{i}$ be the average amount of solid waste needed to be transported in community i per day, and it can be formulated as follows:

(2) $$D_{i}=\lambda_{i}qn_{i},\forall i\in I.$$

#### 2.1.4. Relative Size of the RTS and its Operating Cost

Let $s_{ij}$ be the average amount of solid waste shipped from community i to RTS j per day. $E_{j}$ is the average amount of solid waste transshipped in RTS j per day. $\alpha_{j}$ is the relative size of RTS j, and reflects the proportion of solid waste that will be processed at such a RTS [23]. Then, we have the following Equations (3) and (4):

(3) $$E_{j}=\sum_{i=1}^{I}s_{ij},\forall j=1,2,\cdots ,J;$$

(4) $$\alpha_{j}=\frac{E_{j}}{\sum_{j=1}^{J}E_{j}},\forall j=1,2,\cdots ,J.$$

Note that if $\alpha_{j}$ equals 1, all solid waste will be transferred through one RTS. The operating cost of the RTS is defined as follows: First, a fixed investment $h_{1j}$ that is incurred whenever RTS j is enabled. The fixed investment $h_{1j}$ equals the infrastructure investment of the RTS divided by its expected service time (years). Second, there is a unit variable cost $h_{2j}$, which reflects the varying cost to process the solid waste in the RTS. Let $z_{j}$ be a binary variable that takes the value of 1 if RTS j is opened, then the annual (365 days) operating cost for RTS j can be defined as follows:

(5) $$h_{j}=z_{j}[h_{1j}+365h_{2j}E_{j}],\forall j\in J$$

Note that the linear function $h_{j}$ implies the economies of scale when RTS j is opened. For example, if $z_{j}=1$, $h_{1j}=100,000$, and $h_{2j}=0.5$, then the annual operating cost is $101,825 (100,000 + 365 × 0.5 × 10) if RTS j processes 10 tons of solid waste daily. The unit turnover cost is $27.9 (101,825/3650). However, if 100 tons of solid waste is processed per day in this RTS, the annual operating cost will be $118,250 and the unit turnover cost will be decreased to $3.24 (118,250/36,500). If we combine the total transportation cost with the annual operating cost, the annual operation cost for solid waste collection can be derived.

### 2.2. The Optimization Model

In this subsection, we describe the optimization model for MSW collection in the Nanjing Jiangbei new area. We focused on the decisions of location and allocation of the RTS. To facilitate model formulation in the following context, we provide all notations as follows:

Indexes

   I: Set of communities in the Nanjing Jiangbei new area, i∈I.

   J: Set of RTSs, j∈J. As mentioned above, since we did not know the optimal number of RTS in advance, we used the symbol J to represent the set of possible RTS. In the solution section, we tested all possibilities of this set from one to the maximum number of RTS that could be constructed ($J^{M}$). The optimal number of RTS will be equal to or less than the $J^{M}$ value specified.

   K: Set of landfills in the Nanjing Jiangbei new area, k∈K.

Parameters

   g: Unit transport cost from community i to RTS j.

   δ: Unit transport cost from RTS j to landfill k.

   $\left(a_{i},b_{i}\right)$: Coordinates of community i. 

   $\left(a_{k}^{\prime },b_{k}^{\prime }\right)$: Coordinates of landfill k.

   $D_{i}$: Average amount of solid waste per day in community i.

Variables

   $\left(x_{j},y_{j}\right)$: Coordinates of RTS j.

   $d_{k}(x_{j},y_{j})$: Distance from RTS j to landfill k.

   $d_{i}(x_{j},y_{j})$: Distance from RTS j to community i.

   $u_{ij}$: Binary variable that takes one if RTS j serves community i.

   $z_{j}$: Binary variable that takes one if RTS j is opened.

   $h_{j}$: Operating cost of RTS j.

   $E_{j}$: Average amount of solid waste transshipped in RTS j per day.

   $s_{ij}$: Average amount of solid waste shipped from community i to RTS j per day.

   $t_{jk}$: Average amount of solid waste shipped from RTS j to landfill k per day.

According to the above notations, the optimization model for MSW collection in the new area can be formulated as follows:

(6) $$Min[g\sum_{i=1}^{I}\sum_{j=1}^{J}d_{i}(x_{j},y_{j})s_{ij}u_{ij}+\delta \sum_{j=1}^{J}\sum_{k=1}^{K}d_{k}(x_{j},y_{j})t_{jk}]\times 365+\sum_{j=1}^{J}h_{j}$$

(7) $$s.t.\;\sum_{j=1}^{J}u_{ij}=1,\forall i\in I;$$

(8) $$\sum_{i=1}^{I}s_{ij}u_{ij}=\sum_{k=1}^{k}t_{jk},\forall j\in J;$$

(9) $$u_{ij}\leq z_{j},\forall i\in I;$$

(10) $$\sum_{j=1}^{J}s_{ij}=D_{i},\forall i\in I;$$

(11) $$u_{ij},z_{j}=\{0,1\},\forall i\in I,j\in J;$$

(12) $$s_{ij},t_{jk}\in N,\forall i\in I,j\in J,k\in K;$$

(13) $$x_{j},y_{j}\;\mathrm{are}\;\mathrm{continuous}\;\mathrm{variables}.$$

The proposed model is a mixed integer nonlinear programming model (MINLP). The objective function, Equation (6), minimizes the annual operation costs, which includes the cost of transportation between the communities and the RTSs, the cost of shipping solid waste between the RTSs and the landfills, and the operating cost in these RTSs. Constraint (7) is a single-sourcing constraint that restricts a community’s solid waste to be collected by a single RTS. Constraint (8) is the flow conservation constraint, which ensures that all solid waste will be finally transported to the landfills. Constraint (9) means that the community can only be assigned to the opened RTS. Constraint (10) ensures that solid waste in all communities will be collected. Constraint (11) ensures that $u_{ij}$ and $z_{j}$ are binary variables. Constraint (12) requires that all flow variables, $s_{ij}$ and $t_{jk}$, are non-negative integers. Finally, $\left(x_{j},y_{j}\right)$ in Constraint (13) take any coordinates in the new area.

The above model simultaneously decides how many RTSs should be opened and where to locate them. Furthermore, it determines the relative size of each RTS and the annual operation costs for different scenarios. Note that our model is an extension of MWP and thus is a nondeterministic polynomial-time hard (NP-hard) problem. The main difficulty in solving NP-hard problems arise because the objective function is not convex and has a large number of local minima. Therefore, we need to design an effective algorithm to solve the proposed model.

## 3. Solution Procedure

### 3.1. Model Modification

To solve the proposed model, we first modified it as an equivalent MINLP model and then designed an efficient algorithm to solve it. Note that the term $s_{ij}\times u_{ij}$ in the objective function is non-linear because it involves the multiplication of two decision variables. To avoid the complexity of such terms, we introduced an auxiliary variable $\theta_{ij}$ and defined it as follows:

(14) $$\theta_{ij}=s_{ij}\times u_{ij},\forall i\in I,j\in J;$$

Note that there is a special relationship between the decision variable $s_{ij}$ and the binary variable $u_{ij}$. If $u_{ij}$ equals one, that means that RTS j serves community i. Therefore, $s_{ij}$ is the amount of solid waste shipped from community i to RTS j. Otherwise, if $u_{ij}$ equals zero, which represents that RTS j does not collect the solid waste in community i, thus $s_{ij}$ should be equal to zero. In order to guarantee that the value of $\theta_{ij}$ is equal to the exact value of $s_{ij}$, we can linearize the related constraints by using McCormick inequalities [24]. Let M be a large positive number, and the following constraints should be added to the optimization model:

(15) $$\theta_{ij}-u_{ij}M\leq 0,\forall i\in I,j\in J;$$

(16) $$\theta_{ij}-s_{ij}\leq 0,\forall i\in I,j\in J;$$

(17) $$s_{ij}-\theta_{ij}+u_{ij}M\leq M,\forall i\in I,j\in J;$$

(18) $$\theta_{ij}\in N,\forall i\in I,j\in J.$$

Accordingly, Equation (8) can be rewritten as:

(19) $$\sum_{i=1}^{I}\theta_{ij}=\sum_{k=1}^{k}t_{jk},\forall j\in J.$$

Similarly, let $\psi_{j}=z_{j}\times E_{j}$ and we can linearize the term of $\sum_{j=1}^{J}h_{j}$ in the objective function. Correspondingly, the following constraints should be added to the model.

(20) $$\psi_{j}-z_{j}M\leq 0,\forall j\in J;$$

(21) $$\psi_{j}-E_{j}\leq 0,\forall j\in J;$$

(22) $$E_{j}-\psi_{j}+z_{j}M\leq M,\forall j\in J;$$

(23) $$\psi_{j}\in N,\forall j\in J.$$

Finally, the objective function of Equation (6) can be rewritten as:

(24) $$Min[g\sum_{i=1}^{I}\sum_{j=1}^{J}d_{i}(x_{j},y_{j})\theta_{ij}+\delta \sum_{j=1}^{J}\sum_{k=1}^{K}d_{k}(x_{j},y_{j})t_{jk}]\times 365+h_{1j}\sum_{j=1}^{J}z_{j}+365h_{2j}\sum_{j=1}^{J}\psi_{j}$$

Since $d_{i}(x_{j},y_{j})$ and $d_{k}(x_{j},y_{j})$ are not known in advance, the modified model (Equation (24) together with all constraints) is still a MINLP model. However, once the number and the corresponding locations of these RTSs are given, all distance variables $d_{i}(x_{j},y_{j})$ and $d_{k}(x_{j},y_{j})$ can be determined, and the proposed MINLP model can be addressed as a mixed integer linear programming (MIP) model. In that situation, commercial software such as Cplex and MATLAB can be adopted to solve the MIP model efficiently. The solution algorithm is illustrated in the following subsection.

### 3.2. Solution Algorithm

This subsection developed a solution procedure for the proposed optimization model. Compared with the exact mathematical optimum, a relatively good solution was more in line with the actual needs for MSW collection planning. Therefore, the goal of designing an algorithm in this subsection was to search the solution space efficiently and generate a relatively good solution within a reasonable amount of time (i.e., several minutes or hours, not days or weeks). In this paper, we used the genetic algorithm (GA) to solve the proposed optimization model because if we can code the solution of the problem as a chromosome, GA will then evolve itself until the termination condition is satisfied. Of course, the development of other algorithms for solving this type of problem and comparing the results with our method could also be a direction of future research.

**Figure 2:**
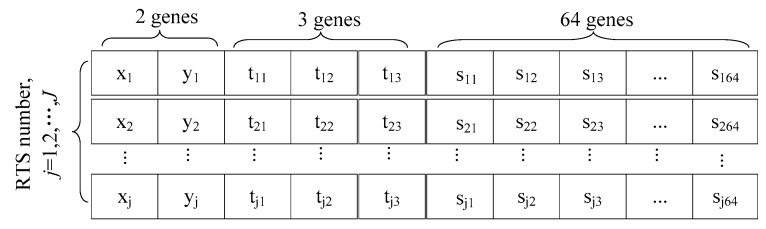
A basic structure of the chromosome.

Figure 2 shows the basic structure of the chromosome for our GA. The first two columns represent coordinates of the RTSs. The following three columns indicate the waste flows between the RTSs and the three landfills. The third part specifies the service relationship between the RTSs and the 64 communities. Since we solved the model for each value of j, if there are j RTSs, a chromosome will include j rows of code. For each chromosome, the coordinates of RTSs are randomly generated. Once the number and the corresponding locations of RTSs are given, the logistics planning among communities, RTSs, and landfills can be addressed as a MIP model and be efficiently solved. Therefore, the latter 67 columns (3 + 64) in each chromosome represent the optimal solution of the corresponding scenario. GA starts by randomly generating a large set of chromosomes. Each chromosome is a feasible solution of the problem. All the initial chromosomes form the initial solutions set, called a “population”.

**Figure 3:**
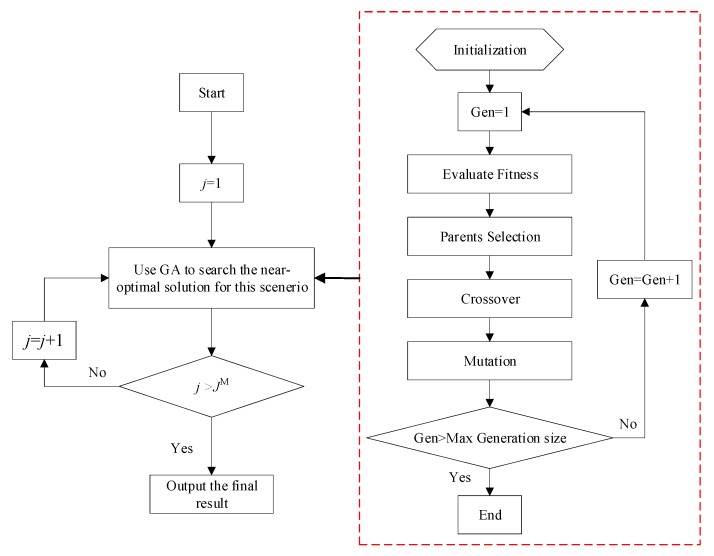
The solution procedure.

It is well known that GA is a kind of mature algorithm and is not sensitive to its parameters including the crossover rate and the mutation rate. The details of GA to solve the MWP model were introduced in [23] and thus we omitted it in this paper. Herein, we briefly introduce the core steps. The fitness of each chromosome is evaluated by calculating the objective function of the proposed optimization model. The roulette wheel selection is used to select the new population. Arithmetical crossover is adopted to explore a better solution between two random points. Finally, a mutation operation helps create offspring that is far from the rest of the population to avoid being stuck at a local optimum. Since we need to test the model for each value of j, it is worth mentioning that our solution procedure hybrids an enumeration rule and the proposed GA. The solution procedure is demonstrated in Figure 3.

## 4. Case Study and Discussion

### 4.1. Data Collection

In our test, coordinates of the three landfills and the 64 communities were obtained in advance. We investigated the number of residents and estimated the average amount of solid waste needed to be collected in each community. The fixed annual investment for a RTS was estimated as $250,000. The unit cost for compressing solid waste in the RTS was set as $2 per ton. As mentioned before, the local government uses the TL mode to ship the condensed solid waste from RTSs to landfills, and uses the LTL mode to collect the scattered solid waste from communities to RTSs. We derived the corresponding parameters δ and g as follows: Initially, we obtained the total TL shipping cost and the total LTL expenditures from the local environmental protection department. Then, we obtained the value for g by dividing the total LTL expenditures by the weighted distance traveled. Similarly, dividing the total TL shipping cost by the total TL weighted distance traveled gives the cost δ. Finally, the parameters g and δ are $1.0 and $0.5, respectively. Moreover, parameters for GA were obtained from Reference [23]. The crossover probability was set at 0.6; the mutation probability was set at 0.1; the population size was set at 200; and finally, the maximum iteration number was set at 500.

### 4.2. Test Result Analysis

As introduced in Section 1, our model simultaneously addressed five issues related to the location of the RTSs and allocation of the 64 communities. Obviously, having fewer RTSs can lower the fixed and variable operating costs associated with the RTSs, but also increases both the inbound and the outbound transportation cost as a result of longer delivery distance. As these two costs are inversely related, our optimization objective was to find the best trade-off between the operating costs of the RTSs and the total transportation cost. We detail our test results below.

Initially, Table 1 shows the annual operation cost under different scenarios. The columns of Table 1 represent the RTS number in different scenarios, the fixed cost for establishing the corresponding number of RTS (RTS cost), the transportation cost between RTSs and landfills (TL cost), the logistics cost between communities and RTSs (LTL cost) and the total operation cost. As we can see in this table, the annual operation cost varied with the total number of RTSs and there was an obvious trade-off between the RTS expenditure and the transport spending. For example, when there was only one RTS, the total transportation cost was 8.74 million dollars (the sum of 4.93 and 3.81) and the RTS operating cost was 0.72 million dollars. When there were three RTSs, the total transportation cost decreased to 6.91 million dollars (the sum of 4.68 and 2.23), while the operating costs of the RTSs increased to 1.22 million dollars. Compared to the six scenarios in Table 1, we observed that the three-RTS plan represents the optimal balance because the annual operation cost decreases from the one-RTS solution and reaches a minimum at the three-RTS plan (8.14 million dollars), beyond which it increases again. The test results in Table 1 answered question (1) and the first part of question (5) that were presented in Section 1. The test results also revealed the proportion of different components in annual operation cost. This information can help managers in the Jiangbei new area to effectively allocate the government budget.

Second, Table 2 demonstrates the corresponding locations for the optimal three RTSs in Table 1. The three coordinates represent three sites in the Nanjing Jiangbei new area, respectively. More precisely, the first coordinate (99.5, 132.5) represents No. 132, Laoshan Street in Pukou District, the second coordinate (114, 158.6) denotes No. 246, Xinhua Road in Dachang District, the third coordinate (79.2, 89.3) specifies No. 540, Ningliu Road in Liuhe District. Table 2 also illustrates the relative size of each RTS. Note that the relative size in this study was defined to reflect the corresponding proportion of solid waste assigned to that specific RTS. This definition is significant and can guide local managers to determine the daily processing capacity of each RTS. For example, the results showed that the relative size of RTS-2 was 0.36. This means that 36% of the total amount of solid waste in the Nanjing Jiangbei new area (about 300,000 ton per year) needs to be transferred in such a RTS. Dividing it by 365 days, this means that the processing capacity of this RTS should not be less than 300 ton/day. Finally, the test results in Table 2 have answered questions (2) and (3), which were proposed in Section 1.

**Figure 4:**
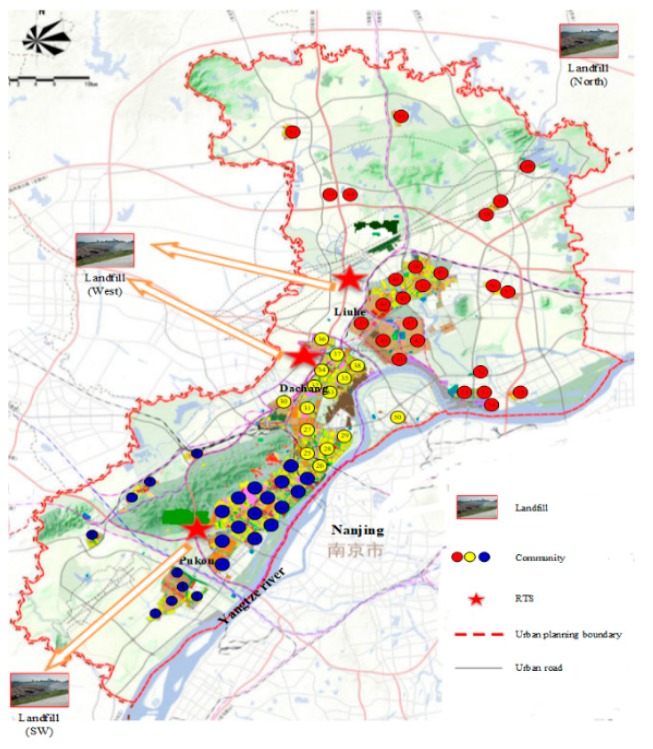
Allocation of each community.

Third, our optimization model also assigned each community to one of the three RTSs to minimize the annual operation costs. We mapped the results in Figure 4. In addition, the relationship between the three landfills and the three RTSs are also described in this figure. The test results in Figure 4 answered question (4), which was defined in Section 1. From this figure, we can see that RTS-1 in Pukou District is mainly responsible for the MSW collection of the southwest region in the Nanjing Jiangbei new area (the blue communities in Figure 4). Then, it transports the compressed solid waste to the landfill-SW due to the minimization in transportation costs. Similarly, RTS-2 in Dachang District provides solid waste collection service for people who live in the center region of the Nanjing Jiangbei new area (the yellow communities in Figure 4). RTS-3 in Liuhe District covers the demand of MSW collection in the north region of the Nanjing Jiangbei new area (the red communities on Figure 4). Both of them deliver the compressed solid waste to landfill-W at the west boundary of Nanjing. Note that landfill-N at the north boundary of Nanjing was not enabled according to our optimal solution. This result may be different from the managers’ intuition. In practice, the landfill-N could be used as an alternative site. Managers can enable it when landfill-W exhausts its capacity.

Finally, Table 3 shows the service level under different scenarios and answers the second part of question (5) that was presented in Section 1. In practice, the percentage of solid waste that can be collected within one hour is an important metric of MSW management, especially at special times, i.e., the Spring Festival in China, when people produce a large amount of solid waste. A popular MSW collection network would be one where the majority of communities are located within 30 km from the corresponding RTS. As can be observed in Table 3, when there is only one RTS, 73.44% of the total amount of solid waste could be transferred in one hour. Among them, only 23.44% of the solid waste could be treated in half an hour and the remaining 50% needs to be addressed in one hour. However, with the optimal three-RTS plan, 98.44% of solid waste in the 64 communities can be collected in one hour and 100% can be treated in 1.5 h. Among them, one can see that the majority of solid waste (71.88%) could be transferred in half an hour, while only a few of them (26.56%) needed to be addressed in one hour. The above results demonstrate that the slight increase in RTS number could extremely improve the corresponding service level. We also noted that the service level was not improved even if there were two more RTSs. The test results in Table 3 are meaningful in practice. Considering that the operating cost for a new RTS is relatively high, managers could decide to adopt our three-RTS plan as the main plan for MSW collection in this new area.

In summary, our model answered the five questions in Section 1. However, we still need to know whether the proposed three-RTS solution remains optimal under different environments. We answered these questions by conducting a sensitivity analysis in the following discussion section.

### 4.3. Sensitivity Analysis

#### 4.3.1. Changes in Unit Transportation Cost

**Figure 5:**
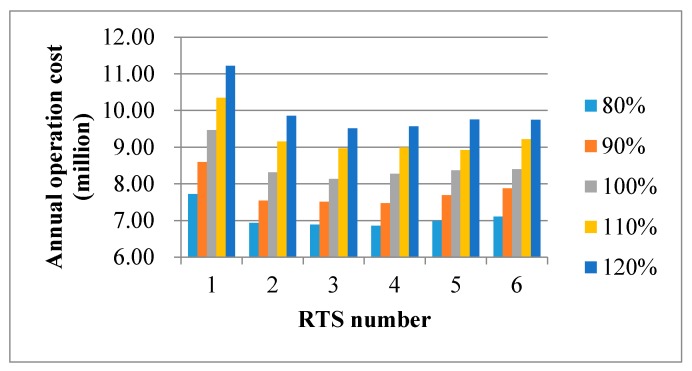
Sensitivity analysis of the unit transportation cost. RTS: refuse transfer stations.

To understand the impact of unit transportation cost uncertainty on the efficiency of the designed network, we conducted a sensitivity analysis with respect to five levels of unit transportation cost (80%, 90%, 100%, 110%, and 120%). Still, we tested each level of unit transportation cost with six scenarios, which corresponded to the possible number of RTS. In total, there were 30 test results in this sensitivity analysis, which are demonstrated in Figure 5. First, we observed that the annual operation cost was proportional to the unit transportation cost in each scenario. The higher the unit transportation cost, the higher the annual operation cost. In practice, fuel types and price may affect unit transportation cost, especially in recent years when fuel prices have frequently fluctuated. Generally, to mitigate the negative impact of increasing transportation cost, it is desirable to adopt more RTSs. However, recalling that the operating cost for a new RTS is relatively high, and constitutes a significant portion of the annual operation costs, thus, the savings in transportation cost from having more RTSs may be offset by the additional RTS cost. A desirable decision hinges on balancing the transportation cost and the RTS expenses. When both costs were taken into consideration, our model recommended the three-RTS solution when unit transportation cost varied from 100% to 120% of the current price. When unit transportation cost decreased (80% or 90%), a new RTS will be suggested. This means that four RTSs need to be opened.

#### 4.3.2. Changes in Costs of a RTS

**Figure 6:**
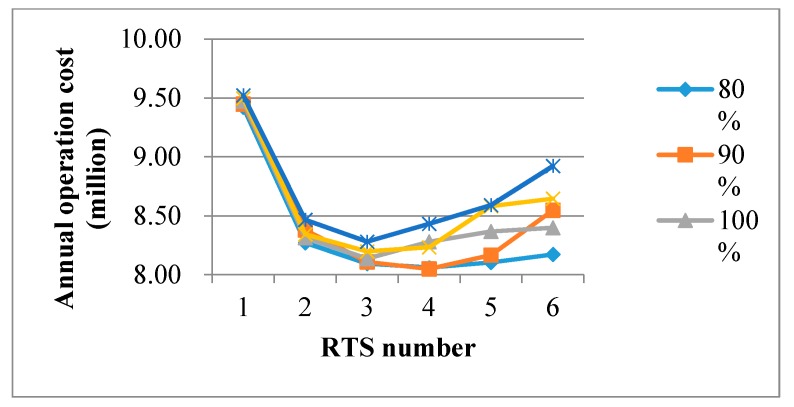
Sensitivity analysis of the fixed annual investment cost in the RTS (refuse transfer stations).

As mentioned in Section 1, the optimization objective in this paper was to find the best trade-off between the total transportation cost and the operating cost of the RTS, while the latter includes the fixed annual investment and the variable processing cost. To examine whether the three-RTS solution remains optimal or not, we tested our optimization model by changing the fixed annual investment from 80% of the current scale to 120% of it (80%, 90%, 100%, 110%, and 120%). Similarly, we tested each level of fixed annual investment with six possible numbers of RTSs, thus we also had 30 test results in the sensitivity analysis. The results are illustrated in Figure 6. On one hand, when there is only one RTS, we observed that the total operation cost was absolutely high, and even the fixed annual investment of RTS was decreased to 80% of its current price. On the other hand, we also observed that when the variation of the fixed annual investment fell in range of (100%, 120%), the three-RTS plan was still recommended. However, when the fixed annual investment of RTS decreased to (80%, 90%), the four-RTS plan would be better.

**Figure 7:**
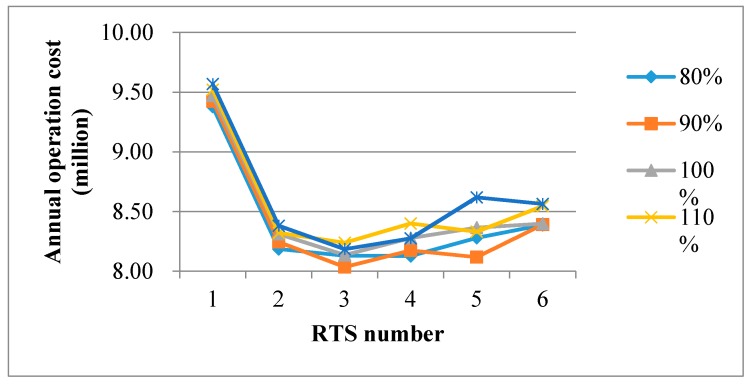
Sensitivity analysis of the unit variable cost in the RTS (refuse transfer stations).

We also constructed 30 scenarios for testing the impact of unit processing cost on the RTS, where the test results are shown in Figure 7. Compared with the sensitivity analysis of the fixed operating costs above, we observed a similar conclusion. When there was only one RTS, the total operation cost was extremely high. The total cost decreased rapidly at first, then increased again after reaching the bottom. The test results in Figure 7 illustrate that the proposed three-RTS plan was still the optimal program when unit variable cost in RTS varied from 90% to 120%. However, when the unit variable cost decreased to 80% of its current price, a four-RTS plan was recommended. Note that the unit processing cost in our optimization model reflects the labor cost, energy consumption, and other influence factors in practice. This makes our test results more significant because the rising labor cost and the strict environmental policies will be the booming challenges in recent years. Managers in local authorities can promptly open and adjust the necessary number of RTSs according to the sensitivity analysis results.

## 5. Conclusions

In this paper, we proposed a MINLP model for designing the MSW collection network in the Nanjing Jiangbei new area. The model simultaneously decided the optimal number of RTSs, identified the appropriate site for each RTS, determined the relative size of each RTS, allocated each community to a specific RTS, and determined the annual operation cost and service level for the optimal scenario as well as other scenarios. The main contributions of this study are summarized below.

First, our optimization model determined the optimal number of RTSs. This model was different to the classic MWP where the number of new facilities to be opened is known in advance. Our model is also different to the classic SCP where the number of new facilities to be opened is totally undetermined. As mentioned in Section 1, our model can be seen as a middle state between the above two extremes. In this study, although we did not preset the number of RTSs, we set a maximum number limitation of RTS. Then, we tested all possible scenarios and finally determined the optimal number of RTSs. These results are significant because it helps to identify the annual operation cost and service level for the optimal scenario as well as other scenarios.

Second, our model simultaneously determined the relative size of each RTS. Note that the relative size of a RTS is proportional to the total amount of solid waste assigned to that specific station, and the operating cost of such a RTS is also related to its relative size. Combined with the first conclusion, we used the optimal result to guide managers to make decisions of how many RTSs should be constructed, where they should be located, and the optimal capacity for each of them. To the best of our knowledge, our method is different from most existing approaches that always preset the capacity of RTS or provide some potential points as the candidate alternatives when modeling the problem.

As for the limitations of this study, we used Euclidean distance to calculate the distance between two points on the planning area. This may alleviate the optimality of the optimal result when applied in practice. Second, the forecasting model for solid waste produced was rather simplified. This may provide an inaccurate forecast at the demand points and thus influence the final network design. Moreover, we used GA in this study to solve the optimization model. This may give an approximate optimal solution within a reasonable amount of time, but not a real optimal result.

Future research can be extended and enhanced in the following directions. Initially, the parameter setting in this study is relatively simple. Further measurements regarding particular parameters need to be conducted to improve the accuracy of the estimated data. Second, we only considered the relationship between the communities, the RTSs, and the landfills in this study. Other recycling infrastructures and processes were not within the scope of this paper. Finally, factors such as environmental emission regulations can be combined into the model, which can help to improve the accurate application of the mathematical model.

## Figures and Tables

**Table 1:** Annual operation costs under different scenarios (unit: million dollars).

RTS Number	RTS Cost	TL Cost	LTL Cost	Total Cost
1	0.72	4.93	3.81	9.47
2	0.97	4.86	2.90	8.73
3	1.22	4.68	2.23	8.14
4	1.47	4.67	2.13	8.27
5	1.72	4.58	2.11	8.42
6	1.97	4.48	2.19	8.65

RTS: refuse transfer stations; TL: full-truckload; LTL: less-than-truckload.

**Table 2:** Location and relative size for the optimal three RTSs.

RTS Number	Location	Relative Size
1	(99.5, 132.5)	0.27
2	(114, 158.6)	0.36
3	(79.2, 89.3)	0.37

RTS: refuse transfer stations.

**Table 3:** Service level under different scenarios.

RTS Number	Service Level for Each Scenario (km)				Total Percentage of Communities Served (h)		
	0–15	15–30	30–45	45–60	1	1.5	2
1	23.44%	50.00%	25.00%	1.56%	73.44%	98.44%	100.00%
2	65.63%	29.69%	4.69%	0.00%	95.31%	100.00%	100.00%
3	71.88%	26.56%	1.56%	0.00%	98.44%	100.00%	100.00%
4	75.00%	23.44%	1.56%	0.00%	98.44%	100.00%	100.00%
5	78.13%	20.31%	1.56%	0.00%	98.44%	100.00%	100.00%
6	85.94%	14.06%	0.00%	0.00%	100.00%	100.00%	100.00%

RTS: refuse transfer stations.
